# Stepchild or Prodigy? Neuroprotection in Multiple Sclerosis (MS) Research

**DOI:** 10.3390/ijms160714850

**Published:** 2015-07-01

**Authors:** Andrea Rottlaender, Stefanie Kuerten

**Affiliations:** Department of Anatomy and Cell Biology, University of Wuerzburg, Wuerzburg 97070, Germany; E-Mail: andrea.rottlaender@uni-wuerzburg.de

**Keywords:** axonal damage, degeneration, multiple sclerosis, neuroprotection, regeneration

## Abstract

Multiple sclerosis (MS) is an autoimmune disorder of the central nervous system (CNS) and characterized by the infiltration of immune cells, demyelination and axonal loss. Loss of axons and nerve fiber pathology are widely accepted as correlates of neurological disability. Hence, it is surprising that the development of neuroprotective therapies has been neglected for a long time. A reason for this could be the diversity of the underlying mechanisms, complex changes in nerve fiber pathology and the absence of biomarkers and tools to quantify neuroregenerative processes. Present therapeutic strategies are aimed at modulating or suppressing the immune response, but do not primarily attenuate axonal pathology. Yet, target-oriented neuroprotective strategies are essential for the treatment of MS, especially as severe damage of nerve fibers mostly occurs in the course of disease progression and cannot be impeded by immune modulatory drugs. This review shall depict the need for neuroprotective strategies and elucidate difficulties and opportunities.

## 1. Introduction

**Figure 1:**
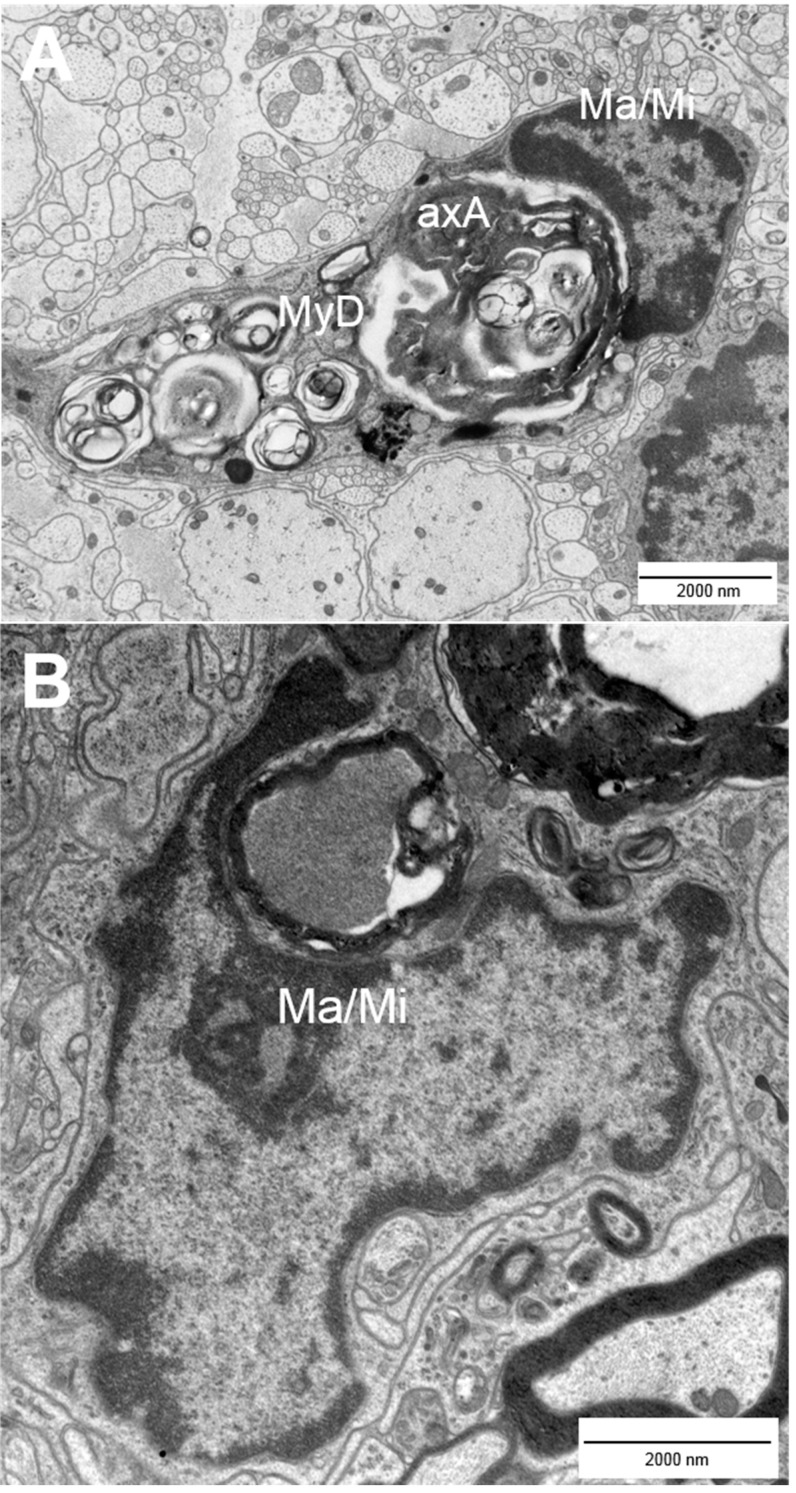
Incorporation of damaged nerve fibers by macrophages/microglia. Activated macrophages (Ma)/microglia (Mi) (**A**,**B**) phagocytose axolytic axons (**A**, axA) and myelin debris (**A**, MyD) within murine EAE spinal cord lesions. EAE = experimental autoimmune encephalomyelitis.

**Figure 2:**
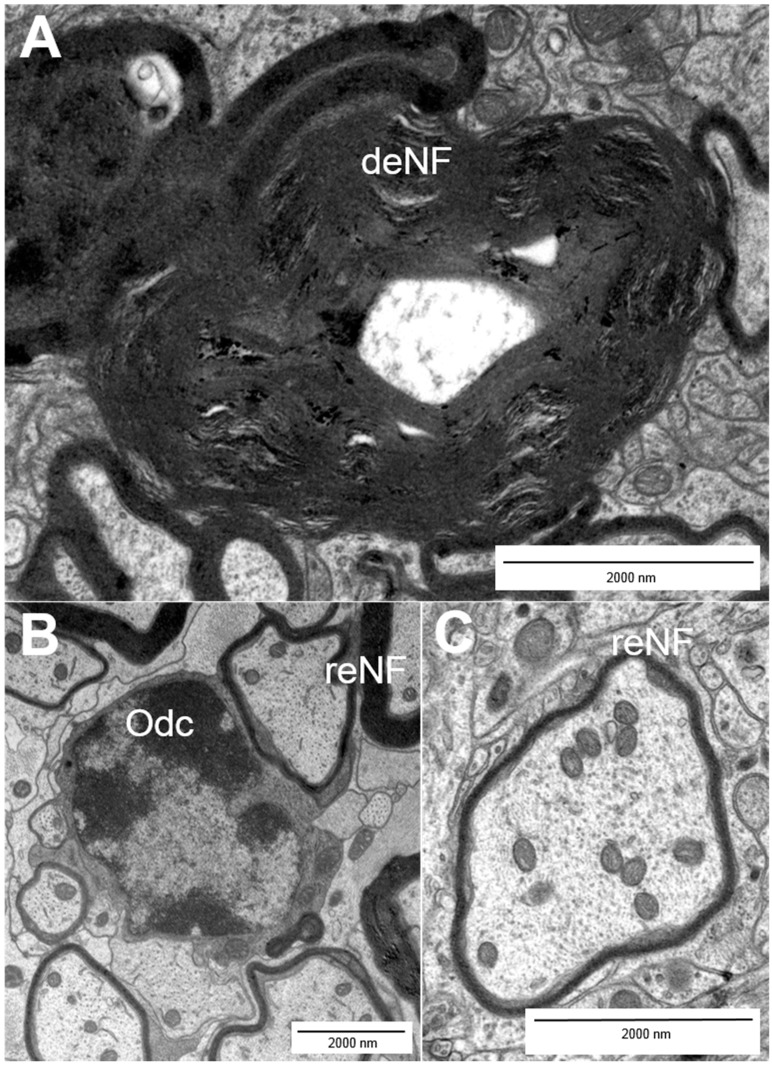
Myelination patterns of nerve fibers: (**A**) demyelinating nerve fibers (deNF); (**B**) olidodendrocytes (Odc); and (**B**,**C**) remyelinating nerve fibers (reNF).

**Figure 3:**
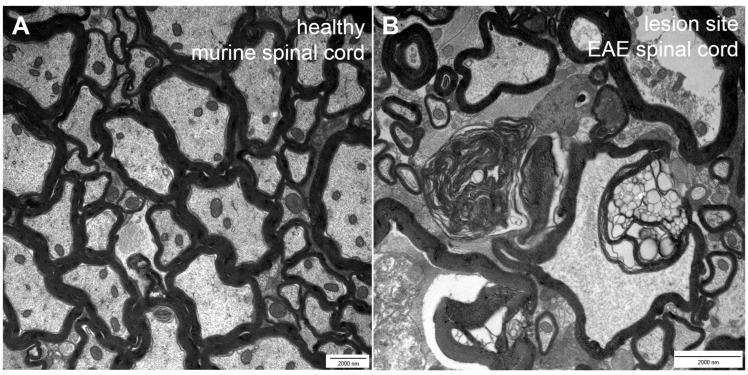
Comparison of healthy (**A**) *versus* EAE lesion (**B**) tissue taken from the murine spinal cord. EAE = experimental autoimmune encephalomyelitis.

Multiple sclerosis (MS) is an autoimmune disorder and the most prevalent disease of the central nervous system in young adults [1,2,3,4]. It is characterized by infiltration, demyelination and axonal pathology [2], leading to multiple neurological deficits. Those range from motor and sensory deficits to cognitive and psychological impairment [5]. Most MS cases (80%–85%) can be described as relapsing–remitting in their clinical course followed by the development of progressive MS with a continuous worsening of symptoms [6,7]. The progressive course of the disease is associated with steadily increasing loss of nerve fibers [6]. Due to substantial neurological impairment, MS often leads to early retirement and thus to immense costs for the social and health care system. Hence, the development of target-oriented therapeutic strategies is of great medical and socio-economic importance. Considering this need, it is surprising that common therapeutic strategies primarily aim at the attenuation of the autoimmune response, while they are unable to eventually prevent disease progression [6]. Currently, there is no cure for MS so that the goal to provide a treatment that allows a persistent prevention of neurodegeneration represents an important area of research and hopefully a valuable addendum to current therapeutic opportunities [1]. Whereas our understanding of the malfunctions of the immune system in MS is steadily improving, little is known about the pathological changes that occur on the level of the nerve fibers. In general, the differentiation of CD4^+^ T cells into the T_H_1 subtype is regarded as main event in the disease pathogenesis [5]. Autoreactive CD4^+^ T cells are thought to become activated in the periphery before they cross the blood-brain-barrier (BBB) and reach the brain tissue where they get reactivated. Subsequently, other cells like B cells, monocytes and CD8^+^ T cells are attracted by cytokines into the inflammatory process. The most common theory about neurodegeneration in MS is that the infiltrating cells—which are the components of the CNS lesions in MS patients—cause edema. Subsequently, demyelination and axonal damage can be initiated by the activation of microglia [8], which release nitric oxide (NO) and show enhanced glutamate production. Glutamate is the major excitatory neurotransmitter and due to over-excitation, nerve fiber injury cascades can be triggered, e.g. by an increase in the intracellular sodium and/or calcium concentration [6,7,9]. In addition, free radicals and cytotoxic CD8^+^ T cells can injure the tissue [4,5,10]. As a consequence of ongoing pathogenesis, changes in the CNS tissue can be detected. These include macrophages/microglia that incorporate or contain myelin lipids in addition to demyelinating and disrupted axons (Figure 1). Injured and nude axons are supposed to cause neurological impairment in patients [4]. Typically, ovoid formation (balloon-like swellings of axons) can be observed in demyelinated lesions. These ovoids are supposed to mark axonal transection and to correlate with irreversible clinical deficits [4,5]. After the acute phase of the disease axonal sprouting can be observed within the injured area as well as in the surrounding tissue and non-harmed oligodendrocytes or oligodendrocyte precursors start remyelinating the demyelinated axons (Figure 2) [5,10]. Figure 3 compares healthy tissue with a lesion site within the murine spinal cord. Of note, the traditional paradigm of inflammation-induced neurodegeneration has recently begun to change since activated microglia and dying oligodendrocytes in the absence of T cell infiltration were observed in acute brain lesions [11,12]. Barnett and Prineas have termed those large areas of apoptotic oligodendrocytes FODOs (“fields of dead oligodendrocytes”) and they were able to detect them in the earliest lesions [11]. Another concept is that MS could be a primary disease of neurons, axons and/or oligodendrocytes that only subsequently triggers an autoimmune reaction in response to neurodegeneration [12,13]. These theories were supported by the rise of imaging techniques that were able to show the occurrence of axonal pathology with disease onset [14,15]. These findings led to the hypothesis that progressive axonal loss starts with the beginning of pathological (inflammatory) processes in MS and that clinical progression is caused by the inability of mechanisms and plasticity to compensate axonal loss [9,12,16]. Interestingly, the observation that nerve fiber pathology is present within early acute inflammatory lesions was already described by Charcot in 1868 [17]. Although the occurrence of neurodegeneration prior to inflammation as mechanism for disease pathology is still a matter of debate, those theories underline that it becomes more and more important to take a closer look at axonal pathology.

## 2. Types of Nerve Fiber Pathology

**Figure 4:**
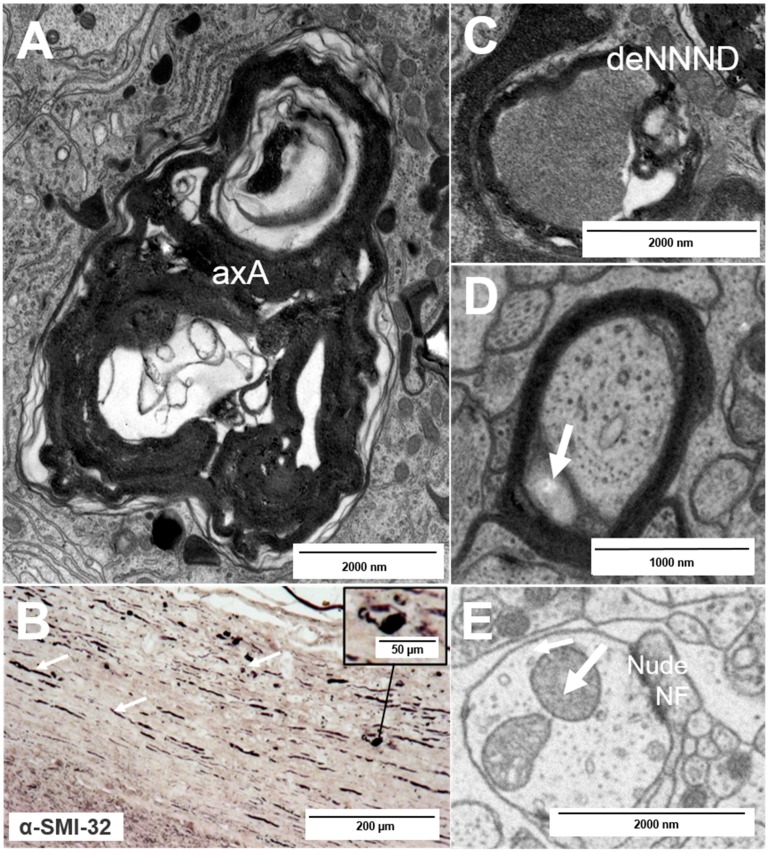
Types of nerve fiber pathology: (**A**) axolytic axons (axA); (**B**) axonal transection as observed after staining for hypophosphorylated neurofilaments (arrows) with typical “ovoid” formation (inset); and (**C**–**E**) fine and early nerve fiber pathology: a decrease in the nearest neighbor neurofilament distance (**C**, deNNND), enlargement of the inner tongue (**D**, arrow) and a nude nerve fiber (nudeNF) with enlarged mitochondria (**E**, arrow).

Nerve fiber injury already occurs during the early phase of inflammatory lesion formation [4,18] and it can also be detected in demyelinated chronic lesions where it is more slow-burning [12,16]. Both chronic and acute axonal injuries are often accompanied by inflammatory cell infiltration, which comprises mainly activated microglia in chronic disease [19]. Generally, neuronal death is enhanced if the affected axon does not have any collateral branches at all or below its lesion site [20]. Whereas axolysis (Figure 4A), axonal transection (“ovoids”, Figure 4B) and axonal loss display final stages of axonal pathology and can be induced by ongoing damage over years [16], several earlier pathological features have been described. Those features are less prominent and can be referred to as “fine” axonal pathology [18]. They comprise a decrease in the distance between individual neurofilaments (nearest neighbor neurofilament distance (NNND)) (Figure 4C), dendritic disruption and an enlargement of the inner tongue (Figure 4D), an increased size or number of mitochondria (Figure 4E), vacuolization of mitochondria and disturbances within the axonal integrity [18,21,22]. In order to quantify normal, demyelinating and remyelinating nerve fibers for histopathology, the g-ratio, which was originally introduced by Guy *et al*., is typically used as an ultra-structural indicator [23]. The g-ratio describes the relationship between the axon and its corresponding myelin sheath. A g-ratio below the optimal range is associated with demyelination and reflective of the ongoing disease process [18]. Nerve fibers with a g-ratio above the optimal range can typically be observed during remission of disease [18,23,24].

## 3. Mechanisms of Neuronal Damage in MS

At present, there is increasing evidence that oxidative damage plays a role in MS and may contribute to degeneration [7,16]. One major source for oxidative stress is microglia activation and associated inflammatory cytokine production (e.g., via NO) [16]. Especially mitochondria are susceptible to oxidative stress and stress in general (e.g., due to increased intracellular calcium levels), which induces changes in membrane permeability, followed by swelling of the mitochondrium and rupture of its membrane [9,12,21]. Additionally, toxic superoxides can be produced by (axonal) mitochondria themselves, especially when they are damaged and induce apoptotic processes via the cytochrome c pathway [12,16]. The activation of caspases is also a common feature in many other neurological diseases [12]. In addition, binding of NO to cytochrome c oxidase can lead to the inhibition of mitochondrial cell respiration [16,21]. Both apoptotic processes and the inhibition of mitochondrial respiration can induce mitochondrial collapse [12]. Mitochondrial damage is often followed by axonal damage even before demyelination occurs [7,16]. One reason for this phenomenon resides in the fact that mitochondria are essential for energy production and that damage of these organelles results in a severe energy deficit of the nerve fiber [4,8,9,16]. This lack of energy supply makes the nerve fiber vulnerable and susceptible to demyelination processes. Once the nerve fiber is demyelinated, its viability is reduced and Wallerian degeneration is more likely to occur [4,12]. Subsequent efforts to remyelinate the nerve fiber frequently result in further energy imbalance. Energy or trophic support cannot be ensured as oligodendrocytes, which normally supply the nerve fiber with trophic factors such as neuregulin or insulin-growth factor 1 (IGF-1), are damaged as well [25,26]. Energy sufficiency is very important for the axon to keep up the sodium and calcium homeostasis. If the homeostasis is disturbed sodium ions accumulate within the axon, leading to malfunction of the sodium-calcium exchanger and consequently to increasing intracellular calcium levels that are cytotoxic, trigger degradation of axonal proteins and further promote axonal death [6,16,27]. In demyelinating nerve fibers, a proper conduction of the respective nerve fiber is lost. As a consequence, sodium channels are upregulated in other areas of the nerve fiber and cause additional sodium influx inducing even higher pathological intracellular ion levels [28]. An increased expression of *Nav1.6*, a sodium channel subtype, was found in injured axons within lesions of MS patients [28]. Intracellular calcium levels can be increased by voltage-gated calcium channels and the stimulation of glutamate receptors, leading to further degradation of axonal proteins. As mentioned before cytokines also play an important role in axonal pathology. On the one hand, they can induce the generation of free radicals leading to oxidative stress and direct damage of nerve fibers [4,5,7,10,16]. On the other hand, they promote the activation of T_H_1 and CD8^+^ T cells. Moreover, they facilitate the communication and attraction of inflammatory cells [4,5,10,16]. Activation of the complement systems can mediate neurodegeneration as well. One major process inducing neurodegeneration is the formation of the membrane attack complex (MAC). This complex disrupts the phospholipid bilayer of the myelin sheath and damages oligodendrocytes. Additionally, the complement system is capable of modulating the T cell response and of inducing cytokine expression and cytolysis [29,30,31]. In response to complement activation so-called anaphylatoxins are released. These factors induce inflammatory responses by attracting neutrophils via their chemotactic properties and by the simultaneous permeabilization of blood vessels [30]. All of these factors favor further damage and are summarized in Table 1.

**Table 1:** Causes of neurodegeneration.

Causes of Neurodegeneration	References
Injury by CD4^+^ and CD8^+^ T cells	[4,5,10]
Free radicals	[8]
Glutamate overload	[8]
Demyelination	[8]
Activation of microglia	[8]
NO	[6,7,9]
Ion accumulation/dysregulation	[6,7,9]
Oligodendrocyte/oligodendrocyte progenitor cell damage	[11,12]
Mitochondrial damage/collapse	[9,12,21]
Others: complement activation, cytokine expression *etc.*	[29–31]

## 4. Physiological Neuroprotection and Remyelination

The term neuroprotection *per se* encompasses the contribution of many factors and processes that are important for neuronal survival. One important process is remyelination, which implies that myelin sheaths and nerve fiber conduction are restored at least partially. Subsequently, lost functions can recover and nerve fibers are protected from (further) degeneration. Usually oligodendrocyte progenitor cells (OPCs) divide in response to processes such as axonal injury, migrate to the site of injury and differentiate into mature oligodendrocytes so that remyelination can take place. Hence, oligodendrocytes and their precursors are crucial for remyelination [16,26,32]. Note that remyelination is not a common but rather a rare phenomenon [16]. Oligodendrocytes show functions beyond remyelination as they are involved in axonal survival [16]. It was shown that even minor changes of oligodendrocytes and the myelin composition can have a drastic effect on the axon such as axonal swelling, swelling of the inner tongue, organelle accumulation and axonal atrophy. Oligodendrocytes are known to transfer trophic factors to the axon [7,16] and release neuronal growth factors (at least *in vitro*) [7,16,20,26]. Additionally, they are suspected to modulate fast axonal transport and the integrity of the axonal cytoskeleton [16,26]. However, oligodendrocytes and OPCs are not the only contributors to neuroprotection. The secretion of neuroprotective factors like brain-derived neurotrophic factor (BDNF), glial cell-derived neurotraphic factor (GDNF), nerve growth factors (NGFs) and the expression of other trophic molecules by immune and glial cells are important for neuroprotective and regenerative processes as well. The capacity to remyelinate decreases with age, probably due to a decreased ability of OPCs in the elderly to differentiate [16]. Cell types/factors that directly contribute to neuroprotection and remyelination are summarized in Table 2. It should be mentioned that there are also cell types that are important for cell communication and migration via their expression profile of receptors or adhesion molecules. These cell types include endothelial cells or vascular components. Endothelial cells, for example, are suspected to respond to and influence the immune process by inducing T cell tolerance [33]. These cell types could therefore play an important role in mediating regenerative processes, but they are acting rather indirectly and are hence not mentioned in Table 2.

**Table 2:** Cell types/factors involved in neuroprotection and remyelination.

Cell Types	References
***T_H_2 cells***:	**[ 12 ]
activation of CNS-resident cells to produce trophic factors (e.g., GDNF by astrocytes), inhibition of T_H_1-mediated toxicity, production of neurotrophins and growth factors like NGFs and IGFs, vascular endothelial growth factors and platelet-derived growth factors	
***Microglia***:	[12,16,20,34]
expression of factors such as NTF, BDNF and GDNF as a “stepping stone” for sprouting axons, release of substances that induce an OPC switch towards a regenerative phenotype (activation) as well as recruitment of OPCs to the lesion site, clearance of myelin debris and lipid recycling (via apolipoprotein E)	
***Astrocytes***:	[12,20]
expression of GDNF, BDNF and factors that induce an OPC switch towards a regenerative phenotype	
***Oligodendrocytes/OPCs***:	[7,12,16,20,26]
expression of trophic factors, support of axonal stability and transport integrity, communication with the axon (e.g., via direct transfer of exosomes to neurons), remyelination	
***Neuron***:	[16]
increased expression of growth-associated proteins (GAPs, like GAP-43) in sprouting nerve fibers and of cell organelle-associated proteins (microtubule/neurofilament-associated proteins)	
***Other factors***:	[6,16,27,28]
strictly regulated ion levels	

Very recently, it was discussed that the CNS has its own lymphatic system, which might be an additional gateway for T cells and other cell types to enter the CNS. This could result in additional pathological mechanisms contributing to the pathogenesis of MS [35].

## 5. Limitations of Regeneration

One major obstacle of remyelination is the formation of a glial scar [20]. This scar is formed by astrocytes in response to CNS damage by hypertrophy and proliferation. This so-called “glia limitans” is a mechanical barrier for the regenerating fibers [20]. Furthermore, several glycoproteins are expressed by astrocytes and other cell types associated with the glia limitans. Those substances inhibit nerve fiber growth to limit neurite expansion under physiological conditions. Under pathophysiological circumstances, remyelination can thereby be inhibited. Another impediment is the insufficient differentiation of OPCs into remyelinating oligodendrocytes and the failure to recruit (enough) OPCs to the lesion site [34]. This insufficient recruitment and further differentiation of OPCs can be caused by the expression of pro-inflammatory cytokines such as TNF-α, which also induce demyelination [34]. Moreover, several proteins are known that are able to inhibit myelination. These include Lingo-1, NOGO-A and PSA-NCAM, which mainly inhibit OPC differentiation and proliferation. Lingo-1 expression is promoted by myelin debris and hence enhanced in lesions [7,16,20]. It also activates RhoA (a GTPase protein) that is normally involved in cytoskeletal dynamics and can mediate pathological damage of the cytoskeleton [9,16,21]. Additionally, the Notch and Wnt signaling pathways have an inhibitory effect on remyelination [7]. All of these factors create a cellular environment that is unfavorable for regeneration and hence remyelination.

## 6. Therapeutic Options for MS

At present, the first-line therapeutic options for MS include the administration of immunmodulatory drugs such as interferon-beta (IFN-β) or glatiramer acetate (GA) [36,37]. Whereas IFN-β mainly aims at attenuating the autoreactive T cell response, GA has some more potential to influence neuroprotection more directly. Its primary effect is based on shifting the T cell response towards a T_H_2 phenotype and hence it can induce the production of neurotrophic factors or anti-inflammatory cytokines by this cell type [36,37]. Moreover, there is some evidence that GA can promote neurogenesis, proliferation and differentiation of OPCs and support remyelination [37]. Some agents are utilized in other neurodegenerative diseases even if they have a different primary indication. In particular minocycline, a tetracycline antibiotic, should be mentioned. Minocycline interacts with neuroinflammatory processes as it attenuates T cell migration and proliferation, microglial activation and inflammatory cytokine production (including inducible NO synthase (iNOS) expression, which is the main producer of NO), glutamate excitotoxicity and cell death [7,35]. In addition, some new strategies that specifically aim at protecting nerve fibers have been developed. For example, damage induced by free radicals could potentially be reversed by the clearance of reactive oxygen and nitrogen species [7,16]. The modulation of NO expression levels could be a further therapeutic strategy. Estrogen and progesterone were shown to protect oligodendrocytes and to promote branching of oligodendrocytes and myelin formation [38,39] by increasing IGF-1 expression of astrocytes [40]. In addition, it was suggested that the insertion of new sodium channels into damaged nerve fiber segments might be capable of re-establishing the conduction along demyelinated axons [12]. Yet another strategy could be to block sodium channels via antagonists like lamotrigine. Indeed, this substance was able to protect axons exposed to nitric oxide [16] and might suppress the activation of innate immune cells, in particular microglia and their migratory activity [16,28]. Unfortunately, the clinical trial failed, as lamotrigine did not protect against initial brain atrophy. However, it should be considered that the outcome was solely rated by the analysis of MRI [6,16]. In addition, only patients with an advanced course of the disease were included in the study. Likewise, the effects of calcium channel blockers are increasingly attracting attention in various *in vivo* and *in vitro* models of CNS injury and the effects of receptor blockade on cellular ion levels, oligodendrocytes and axonal survival are currently being studied [41]. This could be particularly interesting for the modulation of ongoing processes involved in neurodegeneration and subsequently for the chronic stage of the disease. Moreover, several cell types that are involved in CNS regeneration have been implanted into rats. Peripheral nerve segments or olfactory glial cells are both capable of regeneration and once implanted into the lesion site they were able to enhance functional recovery and regeneration of axons [20]. Here, the restoration of the cross-talk between nerve fibers and oligodendrocytes could be a further aspect of increased remyelination [7]. While fetal rat tissue was also implanted into adult animals, regeneration only occurred under optimized conditions (additional trophic support) [20]. An increasing number of studies is dealing with the effects of transplanted neural progenitor and mesenchymal stem cells. Implantation of such cells was able to initiate immunomodulatory responses, oligodendrogenesis and the expression of neurotrophic factors. [20,42]. Remarkably, a reduction of clinical symptoms after transplantation was reported. Right now, some of these cellular therapies are already in clinical trials [20,42]. Experiments to transplant additional or younger OPCs into the lesion site were only partially successful. One reason for this could have been the disseminated distribution of MS lesions [16]. Besides, the correct amount of OPCs needed for efficient remyelination is hard to determine. Ruckh *et al.* [43] were able to show that age-related effects on remyelination were reversible by using heterochronic parabiosis in adult animals, suggesting a possible enhancement of remyelination by OPCs. Inhibitors of OPC differentiation (e.g., humanized monoclonal antibodies against Lingo-1 and other inhibitory proteins) [16,20] and agonists of OPC differentiation, (for example the retinoid X receptor-γ agonist) were successfully applied *in vitro* [7,44]. Schnell *et al.* administered antibodies to proteins inhibiting remyelination (e.g., IN-1 antibodies), which were able to enhance nerve fiber regeneration in rats [45]. Several siRNA-based drugs are currently tested for inducing a functional knock-down. One example is the silencing of caspase-2 expression. Those drugs are presently prepared for use in humans [6]. Another attempt to increase nerve fiber survival has been to stimulate microglia to secrete neurotrophic factors. BDNF and ciliary neurotrophic factor (CTNF) have turned out to be the most promising factors as they have been shown to lead to increased nerve fiber survival [20].

Creating a more favorable environment for regenerative processes could be an important strategy as well. In particular, the regulation of macrophage activity as well as enhancing their phagocytic clearance of myelin debris could be an important step in the generation of more effective regeneration [32]. Other studies aim at modulating CNS damage via the examination of growth factors like IGF-1 and FGF-2, which were both shown to increase neuroregenerative processes and remyelination [1,46]. Frank *et al.* [46] were able to administer IGF-1 to MS patients, but they did not find any significant improvement in MRI endpoints. Of note, however, they did not use advanced MRI techniques like MRI spectroscopy to assess their results. This illustrates the importance of clinical tools and biomarkers to detect and quantify regenerative processes. In general, administration of neuroprotective treatments is limited by several factors. One important aspect is that many substances cannot cross the BBB and are therefore not available for the CNS or only in stages of disease when the BBB is leaky. Other problems are side effects as well as a short half-life [7,20].

## 7. How to Evaluate Therapeutic Benefit?

Therapeutic benefit in MS patients is usually determined by the assessment of the enhanced status disability score (EDSS), the annual relapse rate and MRI lesion activity. One additional parameter is the measurement of brain atrophy, which might reflect axonal and neuronal loss [12,14,15]. Brain atrophy is already used as an outcome measure for clinical or research trials. As brain atrophy is not only due to the loss of nerve fibers and myelin, but also to changes in the tissue fluid and vessel or glial structure, measurements of brain atrophy often lead to conflicting results. The heterogeneity of different patient cohorts (e.g., mixed types of disease or drug treatment) and small numbers of patients have caused inconsistent results. Especially in stem cell therapy only small numbers of patients can be recruited for clinical trails. Development of therapeutic strategies in that area will benefit from larger, multi-centered studies in the future. Hence, additional indicators of therapeutic benefit should be considered. NAA (*N*-acetylaspartate) is a marker of neuronal/axonal dendritic integrity and a reduction in NAA levels in magnetic resonance spectroscopy (MRS) has been shown to correlate with the extent of axonal loss [12,15]. Using NAA-MRS it was shown that axonal loss was only partially reversible and that MS patients displayed a reduction of NAA levels up to 80% [12,15]. In addition, the development of “black holes” (T1-hypointensities) was supposed to correlate with the extent of axonal loss [12,14,15]. However, not all hypodensities progress into chronic black holes, which are associated with axonal and myelin loss [12]. Myelin protection and repair can be observed via T2-weighted relaxation distribution that originates from water in myelin sheaths [12]. Diffusion tensor imaging (DTI) can also be used to characterize nerve fiber pathology. Currently the quantification of neurofilament levels in the cerebrospinal fluid (CSF) and TOB1 expression in resting CD4^+^ T cells are being discussed as potential biomarkers [7]. Today the examination of myelin content, neuronal and axonal integrity and the assessment of brain atrophy as well as pathologic changes within the CNS are possible [12,14,15,16]. However, so far, there are neither satisfying clinical tools to assess the remyelination status of individual patients nor adequate biomarkers [7,16]. As a result, it may still be impossible to actually reveal a truly effective treatment for the prevention of neurodegeneration or the initiation of regeneration, respectively. Even if changes can be observed and quantified by imaging techniques silent axonal loss can occur before symptoms are even detectable in MRI, especially as the brain has the capacity to out-balance neuronal loss or axonal transection. Thus, the development of adequate biomarkers that can be implemented early or even before disease onset are essential.

## 8. Concluding Remarks

After over 140 years since the discovery of nerve fiber pathology in MS lesions by Charcot, there is still no available therapeutic option to attenuate nerve fiber pathology and to prevent progressive axonal loss. An early treatment with neuroprotective agents could attenuate fine and prevent irreversible axonal damage and consequently neurological deficits. Combining anti-inflammatory therapy and neuroprotection should be the most desirable goal for future treatment. Moreover, implantation of progenitor or stem cells could be a promising strategy. Neuroprotective strategies developed for the treatment of MS may also be beneficial for the treatment of other neurological diseases. One major problem is the difficulty in diagnosing MS patients at an early stage of the disease. Usually, the diagnosis is based on excluding other neurological diseases, as MS is heterogeneous. MS lesions and axonal pathology can occur early on even before the first clinical symptoms emerge, which can be explained by the plasticity of the CNS. This is why crucial time for early treatment is frequently lost. The diagnosis of MS relies on the so-called McDonald criteria, which are based upon MRI findings [47]. The McDonald criteria are rated as the gold standard for MS diagnosis, which is attributed to their high sensitivity and specificity. Furthermore, MRI measurements are less invasive than the traditional spinal tap for the detection of oligoclonal IgG bands in the CSF. Even if the revised criteria may enable an earlier diagnosis of MS, time will still pass until the criteria are fulfilled and the final diagnosis is made [47]. This is why the development of additional suitable biomarkers is essential for the development of neuroprotective strategies, which will help to retain axonal integrity from the onset of MS.

### Key Points

√ Multiple mechanisms contribute to axonal pathology: After initial inflammatory damage mechanisms like dysfunction of mitochondria, trophic and ion imbalance, generation of free radicals, oligodendrocyte death, impaired OPC differentiation and remyelination failure lead to ongoing progression of neurodegeneration and disease over time.
√ Oligodendrocytes do not only remyelinate, but also support nerve fibers metabolically and regulate axonal motor proteins, cytoskeletal structure and integrity.
√ Increased intracellular ion levels lead to the disruption of mitochondrial function and degradation of proteins followed by cell death.
√ For the evaluation of therapeutic benefit, further clinical tools and biomarkers are needed and should be combined with common techniques to rate degenerative/regenerative processes and to provide a better overview of the disease processes in MS.
√ The availability of suitable biomarkers will also fuel future studies that are aimed at addressing the need for neuroprotective/-regenerative therapies in MS and other neurodegenerative diseases.
